# RESILIENCE IN HEALTH ADVERSITY: THE ROLE OF COMMUNITY ENGAGEMENT AND TRANSITIONAL THREAT

**DOI:** 10.1093/geroni/igae098.0208

**Published:** 2024-12-31

**Authors:** Ashley Fromenthal, Rebecca Allen

**Affiliations:** University of Alabama, Tuscaloosa, Alabama, United States; University of Alabama, Tuscaloosa, Alabama, United States

## Abstract

Older adulthood is a time of many transitions and oftentimes comes with challenges such as financial difficulties, functional health decline, and a decrease in social support. Together, these challenges may accentuate feelings of loneliness in older adulthood. These challenges may be exacerbated when older adults face threats within their environment such as having housing issues or moving to a “worse” neighborhood. The current study aimed to explore community-based resilience factors for older adults facing transitional threats and functional health struggles centered around Lazarus and Folkman’s Transactional Model of Stress and Coping. Using 2020 data from 4,688 participants in the Health and Retirement Study (HRS), multiple linear regressions were conducted to examine whether the relationship between subjective health and loneliness is mediated by transitional threats, and if social and community engagement moderate the mediating relationship. Results of the regressions show that the overall model is significant F(2,4546) = 78.11, p <.001, and that each of the predictor variables were significantly related to feelings of loneliness. However, results show that the interaction term (engagement x threat) did not explain a significant amount of variance in the model. This may be due to the measurement of these constructs within the HRS. Specifically, trait-dependent measurement of dynamic constructs such as feelings of loneliness and behavioral engagement may not be sensitive enough to detect moderated mediation effects within the context of transitional threats.

